# Network topological model of reconstructive solid-state transformations

**DOI:** 10.1038/s41598-019-42483-5

**Published:** 2019-04-12

**Authors:** Vladislav A. Blatov, Andrey A. Golov, Changhao Yang, Qingfeng Zeng, Artem A. Kabanov

**Affiliations:** 1 School of Materials Science and Engineering, Northwestern Polytechnical University, Youyi West Rd. 127, Xi’an, 710072 PR China; 2 Samara Center for Theoretical Materials Science (SCTMS), Samara State Technical University, Molodogvardeyskaya St. 244, Samara, 443100 Russia; 3 Samara Center for Theoretical Materials Science (SCTMS), Samara University, Ac. Pavlov St. 1, Samara, 443011 Russia; 4 MSEA International Institute for Materials Genome, Jinxiu Rd. 1, Gu’an, 065500 PR China

## Abstract

Reconstructive solid-state transformations are followed by significant changes in the system of chemical bonds, i.e. in the topology of the substance. Understanding these mechanisms at the atomic level is crucial for proper explanation and prediction of chemical reactions and phase transitions in solids and, ultimately, for the design of new materials. Modeling of solid-state transitions by geometrical, molecular dynamics or quantum-mechanical methods does not account for topological transformations. As a result, the chemical nature of the transformation processes are overlooked, which limits the predictive power of the models. We propose a universal model based on network representation of extended structures, which treats any reorganization in the solid state as a network transformation. We demonstrate this approach rationalizes the configuration space of the solid system and enables prediction of new phases that are closely related to already known phases. Some new phases and unclear transition pathways are discovered in example systems including elementary substances, ionic compounds and molecular crystals.

## Introduction

The search for new materials requires a deep understanding of reconstructive processes, which occur at the atomic level and lead to significant changes in the architecture of the substance. With respect to chemical reactions between molecules, such reconstructive processes are always followed by essential reorganization of the solid system, which often results in destruction of the solid system. ‘Single crystal -to- single crystal’ transformations have attracted special attention in the last few years^1^. In this scenario, the substance preserves its crystallinity and the transformation is often followed by reversible or irreversible chemical reactions between molecules. Single crystal-to-single crystal transformations typically occur in organic or metal-organic compounds, in which the mechanism of transformation usually mimics the reaction of isolated molecules. The displacement of atoms during the transition in inorganic solids is less obvious, and special methods are required to model and understand these processes. In the recent Materials Genome Initiative project^2^, the study of reconstructive phase transitions was approached as the identification, mutation and recombination of the underlying architectures (*genes*) of complex materials systems. Such computational approaches could further accelerate identification and screening of the genes within a material, and complement high-throughput (combinatorial) experiments. Since these genes are expected to essentially determine the properties of the material, computational approaches may provide a cost-effective, high-throughput method to shorten the time-to-market for novel materials. Indeed, the task of developing rational models for reconstructive phase transitions goes far beyond the specific fields of solid-state chemistry and physics.

Modeling of solid-state transitions at the atomic level is now possible thanks to the rapid development of density functional theory (DFT) methods over the last 10–15 years. However, several serious obstacles remain, as there are an infinite number of possible transition pathways, and direct scanning of all possible atomic architectures (also termed *configuration space, CS*) requires huge computational effort. Therefore, the development of specialized methods that can rapidly search for the most energetically favorable pathways is crucial. The first attempts to describe reconstructive phase transitions at the atomic level were undertaken long before the DFT era. Mechanisms were suggested for phase transitions in metallic systems between face-centered cubic and hexagonal close packings or body-centered cubic and face-centered cubic structures (martensitic transformations), in ionic crystals between rock salt and zinc blende, and in covalent crystals between diamond and lonsdaleite, among others^3–5^. However, no general scheme was proposed until the 1990’s, when the geometrically shortest pathways were identified manually. Subsequently, geometrical approaches based on group-subgroup relations were developed^6^ and the DFT modeling within the solid-state nudged elastic band (SSNEB) method was devised^7^.

All of the aforementioned approaches only consider the movement of atoms during the transition and do not account for the fact that reconstructive phase transition leads to changes in crystal structure connectivity, when some interatomic contacts are broken and some new contacts are created. Since connectivity is a topological feature of solid structures, then some topological properties should change during a transition. Topological approaches for the study of phase transition mechanisms have been in development since the 1990’s, though these approaches initially described the crystal in terms of abstract 3-periodic 2D surfaces^8^, not in terms of atoms and bonds. Although several phase transitions have been explored using topological approaches, these models have not been widely propagated due to their complexity and a lack of computational tools. Indeed, the limited availability of computational tools for topological approaches is likely related to the fact that no strict universal algorithm has been proposed for the method and each phase transition must be analyzed in a unique manner.

In 2007, we proposed another topological approach for the interpretation of reconstructive phase transitions, which was based on the interrelations between periodic nets and their subnets^9^. However, this approach required improvement as the net-subnet relations did not allow us to find all possible transition pathways or to easily determine the geometry of the transition state. In this study, we present an extension of this approach to a universal topological model of solid-state transformations and provide some examples of the application of this model. We restrict ourselves to phase transitions; no chemical reactions in the solid state are considered and the chemical composition remains the same during the transition.

## Network Topological Model of Configuration Space

**Figure 1:**
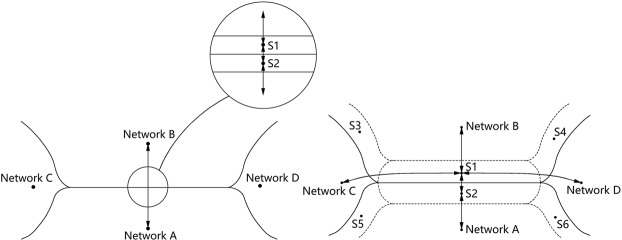
An area of configuration space with four stable network regions (**A**–**D**): (left) a simplified representation with a linear boundary that is formed by two narrow metastable network regions (*S*1 and *S*2); (right) transition pathways from the subnets/supernets to known phases (**A** and **B**) and unknown phases (**C** and **D**).

We consider the configuration space of a crystal system in terms of the network approach. Let us try to assign a particular topology to each point of *CS*. Obviously, there are *CS* regions, where the connectivity can be unambiguously determined (*i.e*., *stable network regions*), as well as intermediate regions, where several topologies can coexist (Fig. 1 left). The existence of several topologies means different nets can be determined for the same atomic configuration depending on the bonding criteria. In particular, some interatomic contacts can be considered as additional bonds or some existing bonds can be ignored, resulting in a *supernet* or *subnet*, respectively. Such configurations can be treated as *metastable network regions* and often provide elongated interatomic contacts, which can be considered or ignored as bonds; it is the configurations that correspond to the subnets or supernets of the nets from stable network regions. Strictly speaking, the boundaries between network regions are fuzzy, since determination of the edges of the nets is somewhat ambiguous; in our model we simplify the *CS* structure and shrink the metastable network regions to the lines between the stable regions (Fig. 1 left).

We propose a rational description of the *CS* within a general network topological model, which is based on representation of the crystal structure within each region as a periodic graph (*net*) and the adjacency of the *CS* regions as the subnet-supernet relations^9^. The model allows us to formulate relatively general statements that govern reconstructive phase transitions and essentially facilitate subsequent, more detailed, quantitative analysis by DFT methods. These statements are:

(i) Any stable *CS* region is described by a unique network topology; the stable regions with different topologies do not cross each other.
(ii) Any reconstructive phase transition is a path, which crosses at least one stable region boundary. In the case of transition *A* → *B*, when nets *A* and *B* are in a direct supernet-subnet relation, the nets immediately transform to each other on the boundary. When an *A* → *S* → *B* transition takes place, the crossing point of the transition path and the boundary is the configuration that corresponds to the supernet-subnet pair *S*. This means that we can equally describe the transition through both the common subnet and common supernet of *A* and *B*. Technically, it is more convenient to consider the supernet as it contains both *A* and *B*, as well as all of their subnets. Several paths may cross the boundary at different geometrical configurations, but at the same network topology. The most favorable path could be determined by the softest vibrational mode of the system.
(iii) Any common subnet of *A* and *B* can be considered as a gene that bears the information common to both nets; different common subnets reflect different properties common to *A* and *B*. Any subnet-supernet transition can be considered as a gene mutation, which leads to a new structure with new properties. This reflects the genetic relations between substances and treats any phase transition as a structure mutation.
(iv) From a topological point of view, the transition *A* → *B* does not require any activation energy, while the transition *A* → *S* → *B* does. This means the former transition does not include a transition state that is topologically different to the *A* and *B* phases.
(v) From a topological point of view, the transition *A* → *B* should be *direct*, *i.e*. pass through a minimal number of boundaries. The higher the number of metastable network regions separating the *A* and *B* phases, the less probable the transition. If several direct pathways exist, the energetically most favorable pathway should be chosen in the subsequent DFT modeling.
(vi) The symmetry of the nets *A* and *B* close to the transition state should coincide with the symmetry of the subnet/supernet and should be as maximal as possible. Since the space group *G* of the transition state is a common subgroup of the space groups of *A* (*G*_*A*_) and *B* (*G*_*B*_), the index of *G* in *G*_*A*_ and *G*_*B*_ should be as small as possible. This also means that *nodality* (*i.e*. the number of non-equivalent nodes/atoms in the subnet/supernet) should not be too high.
(vii) If nets *A* and *B* have no common maximal/minimal subnets/supernets of nodality *k*, then common maximal/minimal subnets/supernets of any higher nodality are also absent. Indeed, if, for example, nets *A* and *B* are both 4-coordinated, and a binodal 4,5-coordinated common minimal supernet exists, then 5,5-coordinated common supernets will also exist (these can be obtained from the 4,5-coordinated supernet by proper addition of an edge). Among the 5,5-coordinated supernets, one can always find a net where the nodes are equivalent, so a uninodal 5-coordinated common minimal supernet should exist. Conversely, if 5-coordinated common minimal supernets do not exist, binodal 4,5-coordinated common supernets will not exist either. This statement is important when looking for common subnets/supernets: if subnets/supernets are absent at a particular nodality, there is no need to search for subnets/supernets at a higher nodality. This also reflects the fact that the higher the nodality, the closer the subnet/supernet to the net. If similarity is not found at a particular level of nodality, there is no need to search for similarity at higher levels.
(viii) Any transition *A* → *S* → *B*, where more than one independent bond is broken/created, i.e. *S* is not a minimal/maximal supernet/subnet of *A* and *B*, can be represented as a sequence of *elementary* transitions *A* → (*S*_i−1_ → *X*_i_ → *S*_i_)→ *B*, where *X*_i_ are intermediate phases and *S*_i_ is a minimal/maximal supernet/subnet of *X*_i_ and *X*_i+1_. This reflects the energy barrier (kinetic) criterion: a minimal number of bonds should be broken/created during the transition. This also means that the detailed structure of the boundary between two regions can be complex and include several metastable transition states (Fig. 1), some of which can be isolated and detected as individual phases. Exploration of the entire set of supernets/subnets is crucial in the case of catalytic chemistry, where we try to decrease some energy barriers to enable preferred chemical reaction pathways.
(ix) The displacements for all atoms during the transition should be as small as possible. From a topological point of view, this means that the atoms of the second coordination shell should be involved in reconstruction of the environment nearest to the origin atom, rather than atoms for further-away coordination shells. This may conflict with the previous condition, so sometimes two possible transitions should be considered: with the minimal/maximal transitional supernet/subnet, but larger atom displacements and the opposite case, when the displacement is minimal, but the number of independent bonds to be broken/created is larger than unity.
(x) In most cases, one can expect each stable network region to contain no more than one minimum potential energy, *i.e*., no more than one energetically stable configuration. Indeed, in most cases, different polymorphic phases have different connectivity, and thus belong to different network regions. Phase transitions within the same network region are either of the first order with low enthalpy, like the *α*-*β*-quartz polymorphism, or second order. The structural differences between such phases are minimal, and obviously it is impossible to find a new architecture in the same network region. This leads to an important improvement of scanning *CS* for new phases using evolutionary algorithms: the initial generation of structures can be formed from the most optimal geometrical embeddings of the networks in each region, *i.e*., each network region can be represented by only one point of *CS*.
(xi) The minimal supernets and maximal subnets could compose a set of promising candidates for the subsequent generation at each step of an evolutionary procedure and hence, speed up the search for new structures. Indeed, being topologically metastable, minimal/maximal supernets/subnets could originate from a variety of pathways leading to new phases (C and D in Fig. 1 right), not only just the *A* and *B* phases that were considered in a particular *A* → *S* → *B* transition. This set allows one to effectively scan the *CS* around the *A* and *B* phases.

Statements (vi) and (vii) essentially restrict the number of subnets/supernets that should be considered for a particular reconstructive phase transition. Strictly speaking the number of different subnets/supernets of a given net is infinite. However, the number of maximal/minimal subnets/supernets is finite at a given subnet/supernet nodality. In turn, the nodality is restricted by statement (vii). So the total number of different subnets/supernets that we have to consider for the initial and final phase is finite and can reach several hundreds for the examples below. However, among these just a few (several) nets are common subnets/supernets for a pair of the structures involved into the reconstructive transition.

Very often, these conditions cannot all be obeyed at once. At least, we can expect that a transition should pass through a maximal/minimal subnet/supernet of rather low nodality and high symmetry. As a result, we can obtain several possible transitions that fit the network model best. The final conclusions on transition probability should be made after appropriate DFT modeling. However, deriving the pathways possible from topological data essentially speeds up the DFT calculations.

We have implemented the principles of the network topological model including the search for supernet-subnet relations in the *ToposPro* code^10^; we have used this code for our topological analysis of all phase transformations. Below, we consider various examples of the application of this model to different classes of substances: elements, ionic compounds and molecular crystals as well as to the prediction of new phases.

## Examples of Model Application

### Diamond-lonsdaleite transition

**Figure 2:**
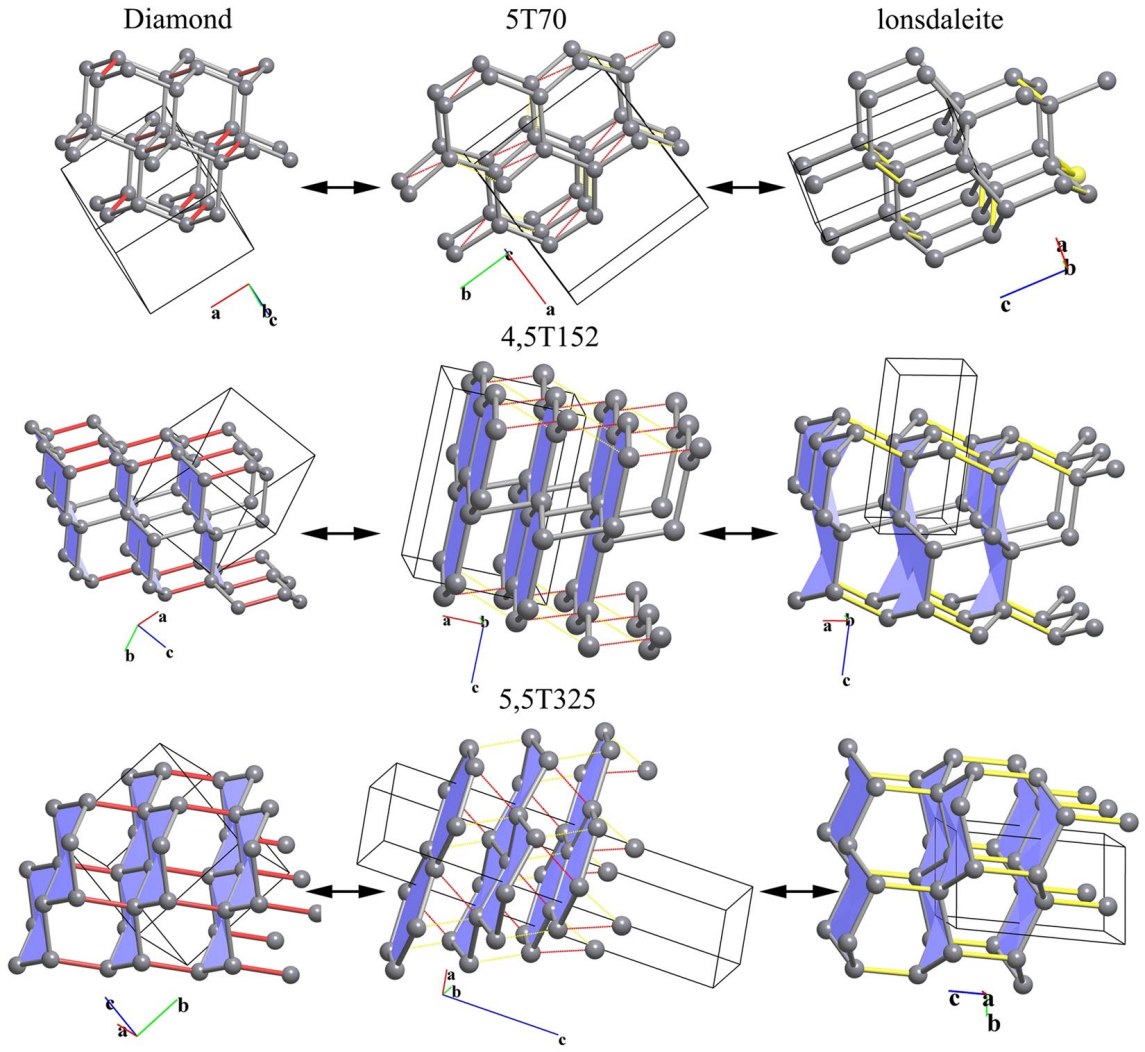
Transition between diamond and lonsdaleite through common, intermediate 5-coordinated, 4,5-coordinated and 5,5-coordinated supernets. The bonds to be broken in diamond and lonsdaleite are highlighted in red and yellow, respectively.

The transition between the diamond and lonsdaleite phases of carbon has been explored many times using different approaches, though only two pathways have been described at the atomic level^5,9^. Both transitions require participation of atoms from the third coordination shell of each carbon atom, and in our terms the transitions pass through common uninodal maximal subnets. Our analysis confirmed these pathways and suggests the corresponding supernet for each subnet (Fig. 2). However, two additional pathways through 4,5-coordinated binodal minimal supernets were identified (Fig. 2); these pathways are the most topologically economic, *i.e*., connectivity is preserved throughout most of the largest part of the initial structure. All transitions involving atoms from the second coordination shell pass through 5,5-coordinated common supernets and the corresponding 3,3-coordinated common subnets, which are not minimal/maximal. The 5,5-coordinated common supernets and 3,3-coordinated common subnets provide the same mechanism of transition through hexagonal layers of a honeycomb topology (Fig. 2), which should lead to graphitization of the system.

### Zinc blende-rock salt transition

Rock salt (NaCl)- and zinc blende (sphalerite, ZnS)-type structures are relatively widespread in inorganic crystals. The respective coordination numbers are six (NaCl) and four (ZnS), and the atoms in each structure are topologically equivalent. A principal difference between the zinc blende-rock salt transition and the diamond-lonsdaleite transition is that NaCl is already a supernet of ZnS. The network model gives a wide range of possible topological transformations in the zinc blende-rock salt transition, but the most symmetrical has *Imm*2 symmetry, for which the subgroup index (12) is four times lower (hence, the symmetry is four times higher) than values previously reported in the literature^4^.

### Graphite-diamond/lonsdaleite transitions

**Figure 3:**
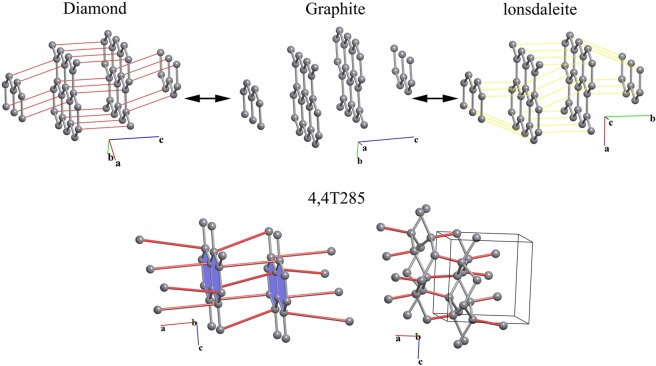
(Top) Transition from graphite (middle, *P*6_3_/*mmc*) to diamond (left, *C*2*/m*) and lonsdaleite (right, *Pnnm*). The additional bonds to be created in graphite are highlighted in red and yellow, respectively; (bottom) a new hypothetical carbon allotrope obtained as a subnet of hexagonal graphite.

The *A* → *B* transitions considered above are characterized by three-periodic *A* and *B* nets. However, the network model also is applicable to transitions between structures with different periodicity, since subnet-supernet relations can be established between any nets. We applied the network model to the transition of two-periodic graphite to three-periodic diamond or lonsdaleite. These transitions have been widely explored both experimentally^11^ and via modeling^12^; however, topological approaches have never been applied. We consider two-layer hexagonal graphite of the *P*6_3_/*mmc* symmetry, which is stable at ambient conditions, and in which all van der Waals contacts between atoms in adjacent layers are possible new edges of the supernet (Fig. 3). Using the network model approach, we found a total of six graphite-diamond transitions with the maximal transition symmetry *C*2/*m*. The movements of the atoms involved in these transitions correspond to the results of a recent molecular dynamics simulation^13^.

### Transitions in ice and silica systems

**Figure 4:**
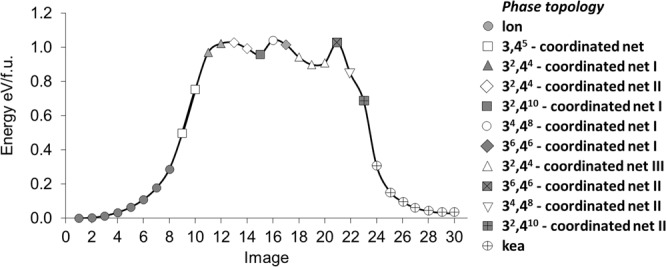
Energy profile for the tridymite ↔ keatite transition.

Ice and silica are chemically quite different, but their structures have many common topological features. In particular, the following pairs are topologically equivalent: ice Ih ↔ tridymite (lonsdaleite topology), ice Ic ↔ cristobalite (diamond topology), and ice III ↔ keatite. Experiments by others have shown the ice Ih ↔ ice Ic and tridymite ↔ cristobalite transitions are feasible and reversible, while the ice Ih ↔ ice III and tridymite ↔ keatite transformations are hindered. These findings were completely confirmed by our network model: the ice Ih ↔ ice Ic and tridymite ↔ cristobalite transitions are topologically simple and pass through a maximal/minimal subnet/supernet like the diamond-lonsdaleite transition (Fig. 2), while the ice Ih ↔ ice III and tridymite ↔ keatite transformations are topologically very complicated and can only be fulfilled through several intermediate topologies (Fig. 4). We did not find any topological route to transform ice II to ice III; indeed, no transition between these phases has been found experimentally.

### Design of new structures

The possible applications of the network topological model extend far beyond interpretation of transition pathways for known phase transitions. As mentioned above, the model can serve as a powerful tool to explore the *CS* and to assist time-consuming methods by revealing the most promising candidates for new phases. Since the minimal supernets/maximal subnets describe the vicinity of a particular topology in the *CS*, by searching for their subnets/supernets, we can find and explore other regions, which may contain new stable topologies. Thus, a subsequent search for all minimal supernets in the graphite network led to discovery of many other possible topologies. Eight of these have already been suggested as possible new carbon allotropes^14^, while the remaining eleven have not been mentioned in the literature. These eleven topologies represent possible *CS* regions around the graphite region; however, only one is metastable while the others decompose back to graphite during their optimization. Using another graphite polymorph with *R*-3*m* symmetry, we obtained one additional new phase. DFT modeling showed that both new carbon allotropes (Fig. 3) have formation energy approximately 0.4 eV/atom higher than diamond.

**Figure 5:**
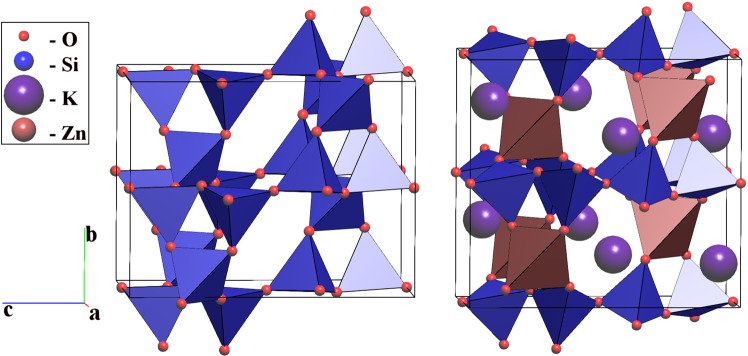
The hypothetical silica phase with **bbi** topology (left) and the crystal structure of K_2_ZnSi_2_O_6_ (right).

The silica system provides another example, in which the transitions from quartz or moganite phases to the keatite polymorph pass through the same intermediate phase with **bbi** topology. Our DFT modelling shows the silica framework with **bbi** topology fits the static and dynamic stability criteria, and lies just 0.05 eV/f.u. higher than quartz at zero temperature and pressure. Although no silica phase with this topology has yet been isolated, our topological databases^10^ contain one example of **bbi**, which was realized in the [ZnSi_2_O_6_]^2−^ framework of potassium phyllo-zincodisilicate K_2_ZnSi_2_O_6_^15^ (Fig. 5).

The approach described above has important advantages compared to other methods for modeling reconstructive phase transitions. In contrast to other geometrical or topological methods^3–6,8^, our approach is based on a rigorous algorithm and is implemented into software (the *ToposPro* code)^10^. The approach is universal, can be applied to any known topology and provides many possible candidates for subsequent DFT estimations. Compared to the DFT SSNEB method^7^, it is much faster and does not require to know the final phase structure; the network topological model generates new phases on its own. Moreover, it does not require enumeration of all possible topologies to find new phases; it establishes genetic relations between already known topologies and uses these relations to predict possible offsprings. In fact, this is a ‘greedy’ type of algorithm for scanning the *CS* for possible local minima. The entire continuous *CS* is replaced by a discrete graph of network topologies and the relations between them. This graph is isomorphic to the net relation graph, in which the relations between the nets and their minimal/maximal supernets/subnets are considered. The net relation graph can be represented as a database and enhanced by applied computational tools for automated scanning of the *CS*^16^. In this work, we have extended this database using the nets discussed in the examples above. Successive exploration of almost 200,000 network topologies stored in our *ToposPro TTD* collection^10^ could essentially enlarge this database in preparation for its combined application with other structure prediction methods.

## Methods

### Network description of crystal structures

The representation of crystal structures as periodic networks originates from a series of work by Wells that started in 1954^17^ and is now the most common approach for the description of crystal structure topology. In this approach, the atoms and/or atomic groups and links between them are represented as nodes and edges of an infinite periodic graph, which is called a *net*. Although the net connectivity (*aka* net topology) cannot be unambiguously determined in some cases, computer algorithms can be applied to generate net connectivity in the same manner as a human expert^18^. In questionable cases, several representations with different topologies can be subsequently enumerated for the same structure^19^. Different crystal structures can have the same net connectivity; in this case they fall into the same *topological type*.

The relations of supernets and subnets are especially important for the interpretation of reconstructive phase transitions^9^. In general, the *subnet* of a net (which is called a *supernet* with respect to the subnet) is formed by subsets of nodes and subsets of edges of the supernet, *i.e*., the subnet can be considered part of the supernet. Since the total number of atoms is preserved during the transition, we only consider the subnets that contain the same number of nodes but that have a smaller number of edges as the supernet. From a chemical point of view, this means that some bonds are broken during the transition from the supernet to subnet, or *vice versa* in the opposite transformation. Obviously, as the number of edges in a net is infinite, each supernet has an infinite number of subnets. Conversely, each subnet has an infinite number of supernets. Their relations can be represented in a graph called *net relation graph*, in which the vertices correspond to the nets, while the edges connect the subnet-supernet pairs^9^.

Any edge in a net relation graph corresponds to a topological transformation of the structure network. From a thermodynamic point of view, the fewer bonds broken during the supernet-subnet transition, the more energetically favorable the transition. Many reconstructive phase transitions can be described by simple supernet-subnet relations; for example, the transition from the rock salt topological type (**pcu-b**) with an atom coordination numbers (CN) six to the zinc blende type (**dia-b**; CN = 4) corresponding to the relation **pcu-b** − **dia-b** in the net relation graph. Hereafter we designate the topological types in accordance with RCSR^20^ or TOPOS^21^ nomenclatures. However, the topological transition could be more complex if the initial and final structures are not in a direct relation, though the transition can always be represented by a path in the net relation graph. For example, the diamond (**dia**) and lonsdaleite (**lon**) nets have the same CN of 4, hence they are neither subnets nor supernets of each other. To pass from **dia** to **lon** some bonds need to be broken and some new bonds created. In terms of net relations, this means a **dia** − *S* − **lon** path, where *S* is a common subnet of both **dia** and **lon**. Obviously the same transition can be represented in an equivalent manner, when one first creates some new bonds and then breaks some old bonds. In this case, *S* is a common supernet of both nets.

Thus, each transition between topologically unrelated crystal structures passes through a transition state, which can equally be described by a common subnet or supernet of the structures. During such transitions, one could expect a minimal number of bonds should be broken and created, which provides the lowest activation energy of the transition state. Thus, *minimal* supernets and *maximal* subnets should be of special interest in this respect. We define a *minimal n-nodal* supernet of a *k-*coordinated net *A*, *i.e*., a *k*, *k*…, (*k* + 1)-coordinated supernet, which contains just one of *n* independent nodes with a coordination number larger by unity than *k*, and hence, just one independent extra bond is created compared to *A*. For example, for a 4-coordinated **dia** net, uninodal (1-nodal) supernets are 5-coordinated; binodal (2-nodal) are 4,5-coordinated; trinodal will be 4,4,5-coordinated, *etc*. Obviously, the larger the nodality *n*, the closer (topologically) the minimal supernet to *A*. The *maximal n-nodal* subnet of *A* is similarly defined, and has (*k* − 1), *k*, *k*…-coordination, *i.e*., only one independent node has a coordination number smaller by unity than *k*, and hence, just one independent bond is broken compared to *A*. The maximal subnets of **dia** are 3-coordinated, 3,4-coordinated, 3,4,4-coordinated, *etc*.

If the crystal structure is described by just one connected network, any new bond being created or broken in the subnet-supernet transition is related to the atoms *A*1 and *A*2 already linked by a sequence of network edges. As such, the number of edges *n* in the shortest sequence determines the ordinal number of the coordination shell of *A*1 (or *A*2), to which *A*2 (or *A*1) belongs. The first coordination shell is composed by the nearest neighbors of a given (origin) atom, which are directly connected to the atom, shells further away include more distant atoms connected to the origin atom through the atoms of the previous shells. The atoms of the further coordination shells change the connectivity of the origin atom during a transition.

Any topology has an infinite number of geometrical realizations, *aka* embeddings into Euclidean space; however, not all realizations fit the network symmetry. The network symmetry is described by an automorphism group (a group of permutations of the network nodes), which is isomorphic to the space group *G* of the most symmetrical embedding of the network^22^. The symmetry of different geometrical embeddings can either coincide with *G* or be a subgroup of *G*. This also provides symmetry relations between the space groups of the subnet and supernet embeddings in the transition state; these must belong to the same Bärnighausen tree^23^, *i.e*., be in a group-subgroup relation. The symmetry of the transition state *G*(*S*) between the *A* and *B* nets cannot be higher than *G*(*A*) or *G*(*B*); *G*(*S*) must be a common subgroup of *G*(*A*) and *G*(*B*). Thus, the corresponding common subnet/supernet *S* of *A* and *B* exists in the transition state in an embedding of the *G*(*S*) symmetry; the maximal symmetry of *S* can be higher, but must be a supergroup of *G*(*S*).

**Figure 6:**
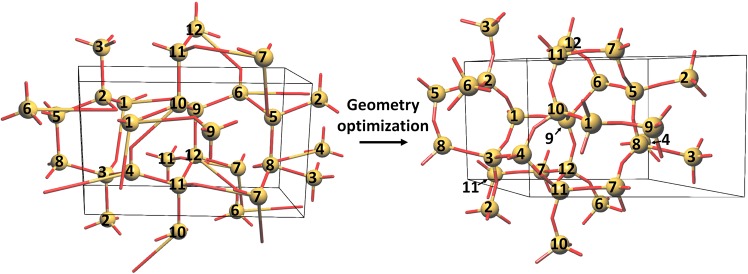
The tridymite phase embedding with the bond system of keatite (left). The optimized structure with the embedding and bond system of keatite (right). Matching atoms have the same numbers.

The common subnet and supernet of the reactant and product structures provide information on which bonds undergo changes during solid-state phase transitions. However, use of this information in DFT modeling of phase transitions for example using the Solid State Nudged Elastic Band (SSNEB) method requires a matching of all atoms. To solve this problem we started by embedding the initial phase with a specified numbering of atoms, but with the bond system corresponding to the final phase; the information on the bonding was obtained from the common supernet (Fig. 6 left). Then we relaxed the system with a molecular mechanics algorithm to obtain the embedding of the final phase (Fig. 6 right).

### Computational methods

Density Functional Theory (DFT) modeling was performed using the Vienna Ab-Initio Simulation Package (VASP)^24^ with the projector augmented-wave method and generalized gradient approximation (GGA). The Perdew-Burke-Ernzerhof (PBE) exchange-correlation functional^25^ was adopted for calculations. For all structures we used a plane-wave kinetic energy cutoff of 600 eV and the energy convergence threshold of 10^−6^ eV. A Γ-centered Monkhorst-Pack^26^ k-points grid was applied everywhere, the force convergence threshold was set to 10^−5^ eV/Å. Several criteria were applied to verify the stability of hypothetical carbon structures: equation of states (total energy vs. volume), elastic stability criteria^27^ and analysis of phonon dispersion curves.

### Phase transition simulations

The search for the most probable initial pathways of phase transition (the correspondence between the end points of the band) were carried out using the network topological model. The minimum-energy path and activation energy of phase transition were calculated via the Solid State Nudged Elastic Band (SSNEB) method as implemented in the Transition State Library for the Atomic Simulation Environment (TSASE)^28^ and VASP code. Both initial and final structures were fully optimized before the SSNEB calculations. For silicon dioxide, we applied an energy cutoff of 600 eV and a 3 × 6 × 4 *k*-points set. Both sets of parameters showed good convergence.

The tridimite-keatite transition was modeled for 30-point SSNEB paths after 75 relaxation steps.

### Diamond-lonsdaleite transition

Sowa and Koch^5^ implicitly applied the network approach to explore the diamond-lonsdaleite transition and used the sphere packing model and group-subgroup relations to suggest a transition through a subnet. They did not characterize the subnet, which has a 3-coordinated **utp** topology, as subsequently shown^9^. We applied the net relation graph concept to find one more pathway through a common 3-coordinated **ths** subnet of **dia** and **lon**^9^. Both transitions require participation of atoms from the third coordination shell of each carbon atom and pass through uninodal maximal subnets of **dia** and **lon**. However, both of those studies had inherent limitations. Sphere packings were only derived down to orthorhombic symmetry at that time, and even now, not all monoclinic packings are found. This explains why the transition through the **ths** subnet, which has a monoclinic symmetry at the transition point, could not be found by Sowa and Koch^5^. Moreover, only uninodal subnets were considered in both papers, so the transitions with higher-nodality nets could not be found. In accordance with the aforementioned features of the network model we explored the most topologically favorable transition pathways.

#### Transitions involving the second coordination shell

The second coordination shell is formed by 12 atoms in both diamond and lonsdaleite. There are no common 5-coordinated supernets, and hence, minimal supernets with higher nodality (see statement (vii) above). Only five different 5,5-coordinated common supernets and the corresponding 3,3-coordinated subnets were found. Each of these provides the same mechanism of transition through the hexagonal layers of a honeycomb (**hcb**) topology (Fig. 2 bottom). Taking into account that such transitions can lead to destruction of the crystal structure, and also that the supernets/subnets are not minimal/maximal, we should consider participation of the next coordination shell.

#### Transitions involving the third coordination shell

Though diamond and lonsdaleite are both 4-coordinated nets, their third coordination shells are comprised of 24 and 25 atoms, respectively, which clearly manifests in topological differences. The search for transitions through uninodal 5-coordinated minimal supernets results in three pathways, two of which - through **sqp** and **sqc2**-5-*C*2/*c* - were mentioned in our previous work^9^; these correspond to transition through a 3-coordinated maximal **ths** subnet. The third pathway passes through the supernet 5T70 (Fig. 2 top), and is equivalent to the transition through the **utp** subnet. Thus, the third coordination shell must be involved to provide the transition mechanisms described in the literature.

Next, we focused on binodal minimal/maximal supernets/subnets, which should be topologically closer to diamond or lonsdaleite than uninodal supernets/subnets according to statement (vii). Two different pathways were found through the 4,5-coordinated minimal supernets 4,5T152 and 4,5T475 and the corresponding 3,4-coordinated maximal subnets **fsg** and **stc**. One such transition is shown in Fig. 2 (middle), in which only some bonds between the hexagonal layers are broken and created (*cf*. Fig. 2 bottom); the network as a whole remains connected. No trinodal minimal/maximal supernets/subnets were found, so the transitions through the 4,5-coordinated supernets are topologically most favorable at this level of consideration.

#### Transitions involving the forth coordination shell

Large atom displacements will be necessary, when atoms from the forth coordination shell are taken into account. Such pathways could be worthy of interest, but only if they pass through a topologically closer transition state than the pathways considered above, *i.e*. trinodal minimal/maximal supernets/subnets should exist at this level. However, we have not identified such supernets/subnets, suggesting these pathways are restricted by previously described pathways. Moreover, this indicates that the closest topological similarity between diamond and lonsdaleite only exists at the level of 4,5-coordinated supernets (or 3,4-coordinated subnets).

### Zinc blende-rock salt transition

The topological similarity of the atoms in rock salt (NaCl) and zinc blende (sphalerite, ZnS) means their topologies can be described by uninodal nets, i.e., primitive cubic (**pcu**) and diamond (**dia**), respectively. Their description as quasi-binodal **pcu-b** and **dia-b** nets does not affect the overall connectivity, but decreases symmetry from *Pm*-3*m* (**pcu**) to *Fm*-3*m* (**pcu-b**) and from *F*-43*d* (**dia**) to *F*-43*m* (**dia-b**). This transition was considered from a topological point of view by Sowa^4^ within the same group-subgroup approach for sphere packing. As a result, a transition through a common *P*3_2_ subgroup, which has an index of 48 for *Fm*-3*m* and 24 for *F*-43*m*, was suggested.

**Figure 7:**
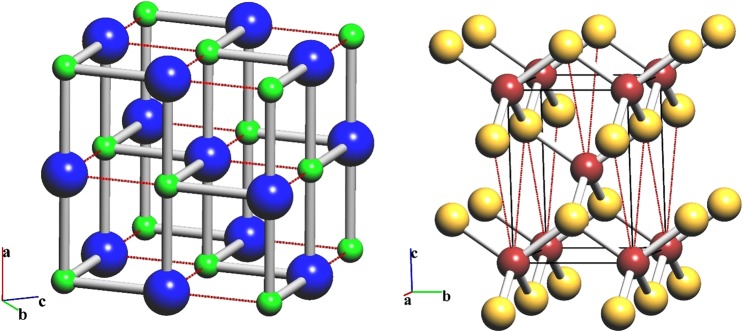
Transition between the rock salt and zinc blende structure types in space group *Imm*2. The bonds to be broken in rock salt or to be created in zinc blende in during the transition are marked in red.

Application of the network model gives a wide range of possible topological transformations from **pcu-b** to **dia-b** in space symmetries *Imm*2, *Pnn*2, *Cm*, *C*2, *P*3_1_/*P*3_2_, *Cc*, *Pc*, *P*2_1_ and *P*1, with a range of subgroup indices from 12 to 48 with respect to *Fm*-3*m*, though the most symmetrical transition state corresponds to *Imm*2 (index 12). This symmetry is four times higher than *P*3_2_; in particular, it leads that both bonds to be broken/created at each atom during the transition (Fig. 7) are symmetrically equivalent in *Imm*2, but not in *P*3_2_. Obviously, the *Imm*2-symmetry transition should be preferred, as it occurs in one stage (both bonds are transformed at once).

### Graphite-diamond/lonsdaleite transitions

Since the 3-coordinated graphite net with the hcb (honeycomb) topology is a minimal uninodal subnet of both the **dia** and **lon** nets, **hcb** ↔ **dia** and **hcb** ↔ **lon** should be direct transitions. Indeed, the set of hcb subnets is one possible transition state for the **dia** ↔ **lon** transition (Fig. 2 bottom). The possible pathways depend on the positions of the **hcb** layers with respect to each other. Considering all van der Waals contacts between the atoms in adjacent layers of two-layer hexagonal graphite as possible new edges of the supernet (Fig. 3 top), we found a total of six graphite-diamond transitions with transition symmetry *C*2/*m* and index six in *P*6_3_/*mmc* and *P*3_1_21/*P*3_2_21, *C*2/*c*, *P*2/*c*, *C*2, or *P*-1, all index 12. The graphite-lonsdaleite transitions can have transition symmetry *Pnnm* or *Cmc*2_1_ (both index six), *P*6_1_/*P*6_5_, *Pnna*, *Pca*2_1_, *P*2_1_2_1_2, *P*2_1_/*c*, *Cc*, or *P*2_1_ (all index 12). The most symmetrical transitions are shown in Fig. 3 (top).

### Hypothetical carbon allotropes

**Figure 8:**
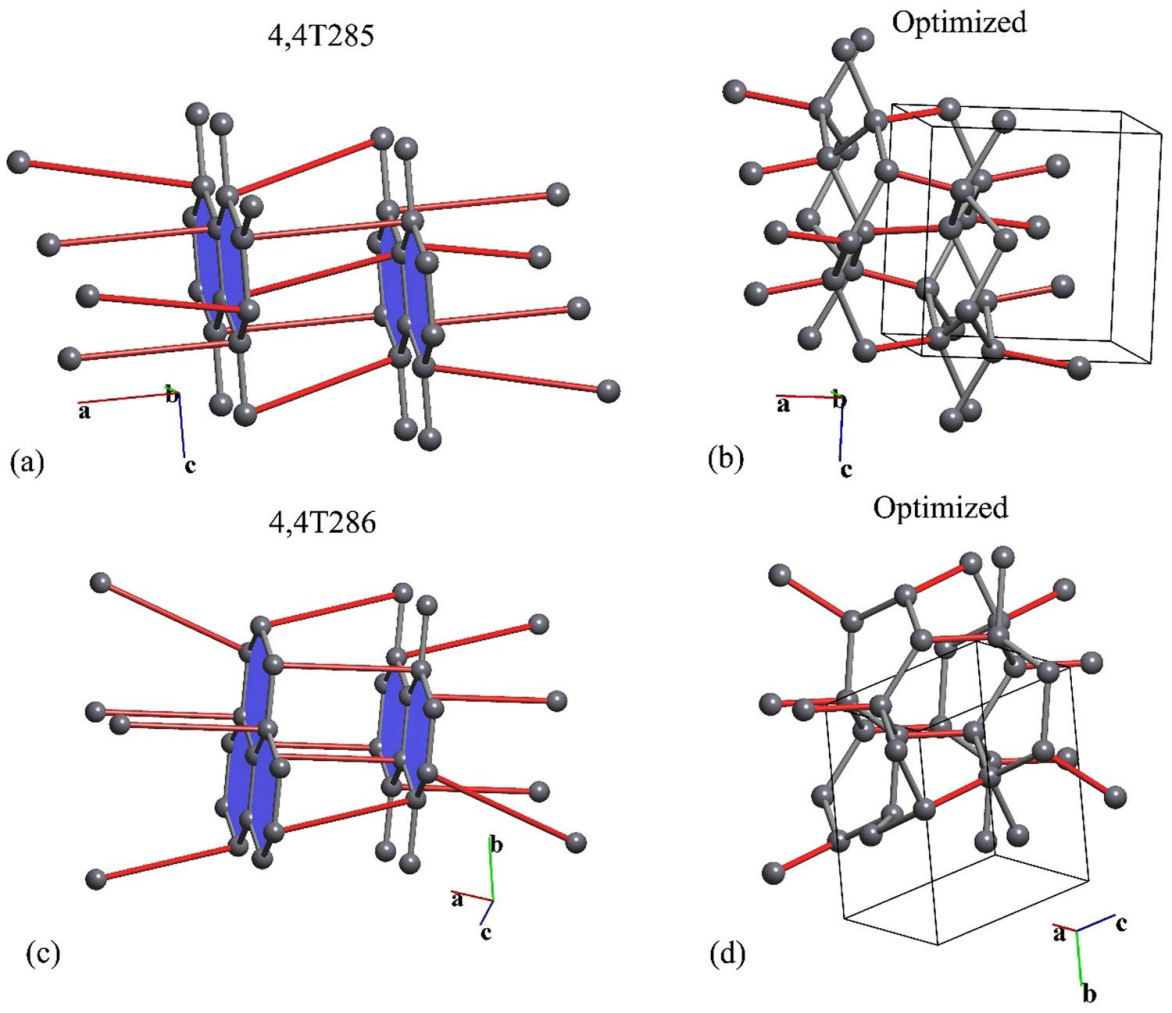
The new networks, named 4,4T285 (**a**) and 4,4T286 (**c**), and structures of the corresponding graphite polymorphs (**b,d**) after optimization.

Since new carbon allotropes are usually obtained by applying high pressure to graphite phases^11^, we can expect the subnet-supernet relations that involve the graphite networks should be crucial for the prediction of as-yet unsynthesized carbon structures. We explored the vicinity of the network *CS* regions for two known graphite phases: hexagonal (*P*6_3_/*mmc*) and rhombohedral (*R*-3*m*). For this purpose we generated all 4-coordinated supernets of these graphite networks in all *P*6_3_/*mmc* and *R*-3*m* space subgroups. After determination of the supernet topologies, we revealed eight previously known hypothetical carbon allotropes, which were already included in our SACADA database^14^, as well as eleven 4-coordinated networks that have not been reported before. Two of the new networks are stable according to our DFT modeling and have been deposited in the *ToposPro TTD* collection under the names 4,4T285 and 4,4T286; these networks be considered as possible new carbon allotropes (Fig. 8). These phases are insulators, about 0.4 eV/atom higher than diamond, and have excellent mechanical properties similar to those of diamond; for more details, see the Supporting Information.

### Ice phases

The phase diagram of water is extremely complicated: twenty phases have been reported at different temperatures and pressures; many of these are metastable. Moreover, in most cases, the transitions between the ice phases, which have a common boundary on the phase diagram, were not detected experimentally. One explanation could be that reconstruction is geometrically and/or topologically hindered in such cases. Let us consider a triplet of phases: ice Ih, which is stable at normal conditions, and ice II and III, which can be obtained from ice Ih at higher pressures^29^. However, the direct transition between ice II and III was not detected. Within the network topological model, this means the corresponding nets cannot easily transform into each other in contrast to the ice Ih ↔ ice II and ice Ih ↔ ice III pairs.

#### Ice Ih ↔ ice II transition

**Figure 9:**
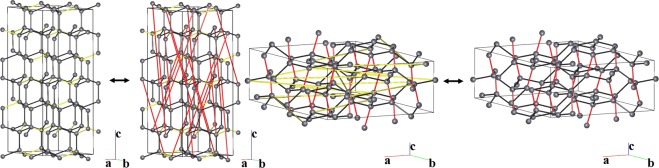
Transition between ice Ih (left, **lon** topological type) and ice II (right, **ict** topological type) through an intermediate supernet (middle). The bonds to be broken in ice Ih and ice II are highlighted in yellow and red, respectively. The water molecules are represented by their centers of mass.

Topologically ice Ih, the system of water molecules with the **lon** topology, can be easily transformed into ice II (*R*-3*c* space symmetry, **ict** topological type) through a common 3-coordinated subnet of **pcu-h** topology. The highest symmetry of the transition state (*R*-3) is close to ice II symmetry. The corresponding uninodal 5-coordinated common supernet has a unique topology, which has not been described before (Fig. 9).

#### Ice Ih ↔ ice III transition

**Figure 10:**
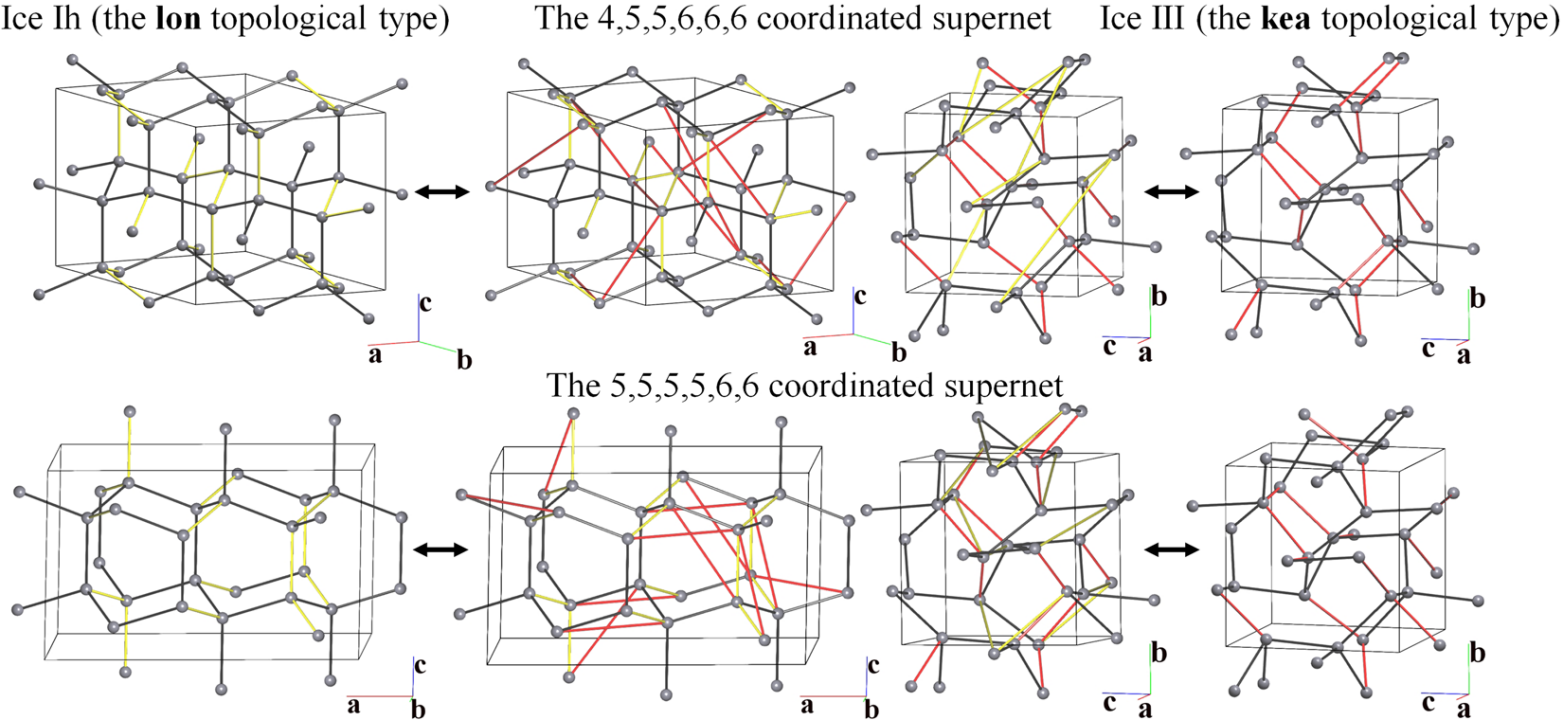
Transition between ice Ih (left, **lon** topological type) and ice III (right, **kea** topological type) through the intermediate supernets (middle) with 4,5,5,6,6,6 (top) and 5,5,5,5,6,6 (bottom) coordination. The bonds to be broken in ice Ih and ice II are highlighted in yellow and red, respectively.

The topology of the network of water molecules in ice III is similar to that of a high-pressure phase of a silica polymorph keatite (**kea**). Two transitions are possible between **lon** (ice Ih) and **kea**, and both of these pass through low-symmetrical (*P*2_1_) subnet-supernet pairs with six non-equivalent nodes, which have 4,5,5,6,6,6 and 5,5,5,5,6,6 coordination in the supernets (Fig. 10).

Therefore, topologically the ice Ih ↔ ice II transition is much easier than the ice Ih ↔ ice III transition. This conclusion has been confirmed experimentally: the former transition is quite common, while the latter requires special conditions^29^.

### Silica phases

Topological features can be common to relatively different crystal structures, and this allows us to reveal similarities in the corresponding transformations. The carbon and ice phases find analogs among silica polymorphs. The topologies within the groups diamond – ice Ic – cristobalite, lonsdaleite – ice Ih – tridymite, ice III – keatite are the same; however, silica also possesses unique phases, quartz and coesite that do not exist in carbon or water systems. This means that some parts of the corresponding *CS*s are similar, while others have distinct differences. One can expect that the topological mechanisms of transformations between these pairs of similar phases should also be similar. Indeed, it is known that a silica high-pressure phase, keatite, can transform into high-temperature cristobalite and tridymite in the same way ice III transforms to ice Ih. We can expect that the transition states are also topologically the same, and for the keatite – tridymite transition the transition states are described by 4,5,5,6,6,6- and 5,5,5,5,6,6-coordinated supernets. These polynodal supernets are obviously not minimal and according to statement (viii), the transitions through these supernets can be represented as a sequence of elementary transitions. Depending on the order in which the bonds are broken/created in such transitions, different transition routes can be obtained and different intermediate networks can be isolated. Importantly, some of these networks describe other silica minerals (quartz and moganite):

$$\begin{array}{rcl}{\rm{Tridymite}}\,({\bf{l}}{\bf{o}}{\bf{n}}) & \to  & {\rm{quartz}}\,({\bf{q}}{\bf{t}}{\bf{z}})\to {\bf{b}}{\bf{b}}{\bf{i}}\to {\rm{keatite}}\,({\bf{k}}{\bf{e}}{\bf{a}});\\ {\rm{Tridymite}}\,({\bf{l}}{\bf{o}}{\bf{n}}) & \to  & {\rm{moganite}}\,({\bf{m}}{\bf{o}}{\bf{g}})\to {\bf{b}}{\bf{b}}{\bf{i}}\to {\rm{keatite}}\,({\bf{k}}{\bf{e}}{\bf{a}}).\end{array}$$

Both quartz and moganite are topologically closer to tridymite (than keatite) and can be obtained from the tridymite network by one elementary transition. This relation and also the close relationship between quartz and moganite have been experimentally confirmed: the phase transition between quartz and tridymite is enanthiotropic, while moganite is often present in quartz rocks and described as a quartz derivative^30^. Moreover, the transition of quartz or moganite to keatite passes through the same intermediate phase with **bbi** topology. The network configuration space model indicates **bbi** topology plays a key role in all reconstructive transitions involving the keatite phase as it is topologically close both to keatite and to other more common silica phases.

Our DFT SSNEB modeling, which was performed for the route described by the 5,5,5,5,6,6-coordinated supernet, confirms this complicated transition pathway, which has two microminima. In total, the transition passes through ten topologically inequivalent metastable network regions (Fig. 4); eight of the 24 inequivalent Si-O bonds are broken and new bonds are formed during the transition.

## Supplementary information

Supplementary Info

Tridymite <-> keatite transition

## Data Availability

The data that support the findings of this study are available from the corresponding authors (V.A.B. and Q.Z.) upon reasonable request.
