# Supplementary material for: Network topological model of reconstructive solid-state transformations
Source: Sci Rep. 2019 Apr 12;9:6007. doi: 10.1038/s41598-019-42483-5 (PMC6461690; doi:10.1038/s41598-019-42483-5)
Supplement: Supplementary file 1 — Supplementary Info [file 41598_2019_42483_MOESM1_ESM.pdf]

Network topological model of reconstructive solid-state transformations

Vladislav A. Blatov,[1,2,3*] Andrey A. Golov,[2,3] Changhao Yang,[1] Qingfeng Zeng,[1,4*] Artem A. Kabanov[2,3]

[1]School of Materials Science and Engineering, Northwestern Polytechnical University, Youyi West Rd. 127, Xi'an 710072, PR China

[2]Samara Center for Theoretical Materials Science (SCTMS), Samara State Technical University, Molodogvardeyskaya St. 244, Samara 443100, Russia

[3]Samara Center for Theoretical Materials Science (SCTMS), Samara University, Ac. Pavlov St. 1, Samara 443011, Russia

[4]MSEA International Institute for Materials Genome, Jinxiu Rd. 1, Gu'an 065500, PR China

Supporting Information

Table S1 | Calculated elastic constants (GPa) of the new carbon allotropes (4,4T285 and 4,4T286) and SiO_2-**bbi** phase.

Phase	C11	C22	C33	C44	C55	C66	C12	C13	C15	C23	C25	C35	C46
4,4T285	95.4	103.4	118.7	-	-	-	8.6	13.2	-	13.4	-	-	-
4,4T286	113.5	101.9	126.6	34.2	50.5	49.1	0.5	6.1	0	14.4	0	0	0
**bbi**	115.3	56.3	135.5	43.1	11.1	32.7	56.7	48.47	-	0.27	-	-	-

Table S2 | Calculated bulk properties of diamond, 4,4T285 and 4,4T286 carbon allotropes.

Allotrope	Density, g/cm^3	E_g, eV (GGA-PBE)	Relative energy, eV/atom	B, GPa	G, GPa	H, GPa
**dia**	3.530	4.20	0	482	546	89
4,4T285	3.449	2.61	0.4747	428	465	77
4,4T286	3.446	2.12	0.5347	416	474	83

Table S3 | Calculated bulk properties of quartz and SiO_2-**bbi** phase.

#	Sp.Gr.	Density, g/cm^3	E_g, eV (GGA-PBE)	Relative energy, eV/f.u.	B, GPa	G, GPa	H, GPa
quartz	P 32 2 1	2.522	5.39	0	34	43	21
**bbi**	$C\ 2\ 2\ 2_1$	2.081	2.61	0.05	48	25	3

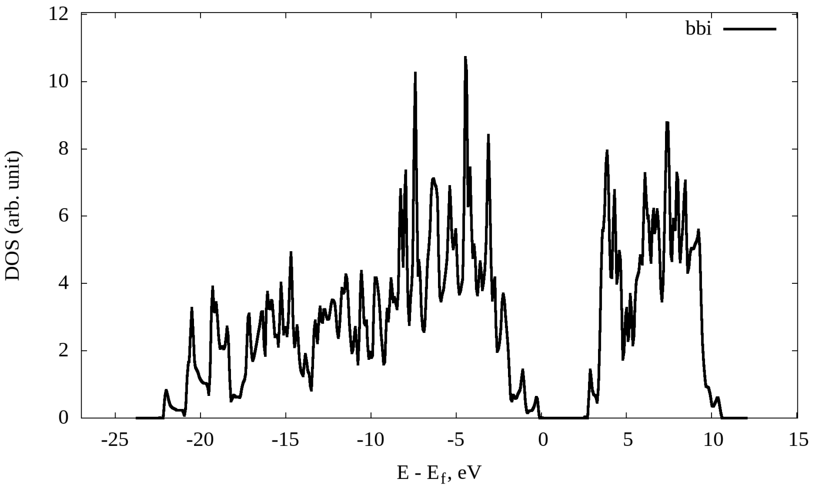

Figure S1 | Density of states plot for the SiO_2-**bbi** phase.

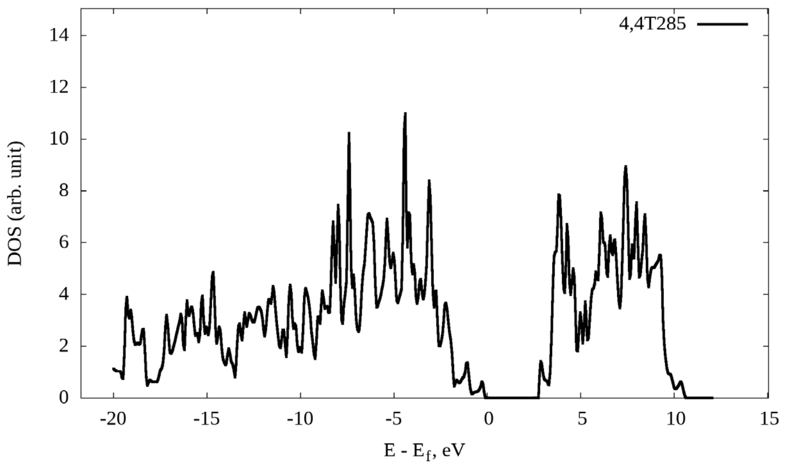

Figure S2 | Density of states plot for 4,4T285 carbon allotrope.

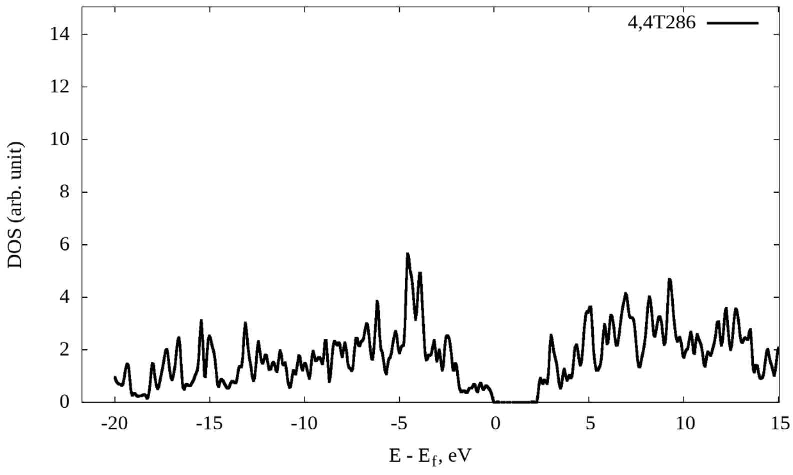

Figure S2 | Density of states plot for 4,4T286 carbon allotrope.

Structural data on new 4,4T285 and 4,4T286 carbon allotropes and SiO_2-**bbi** phase.

4,4T285 carbon structure (.vasp file):

```
4,4T285
   1.00000000000000
     4.4243136943374228    0.0000000000000000   -0.0000000000000000
     0.0000000000000000    4.7568139864781678    0.0000000000000000
     0.0000000000000000    0.0000000000000000    4.3953709404295571
   C
    16
Direct
  0.1733269221490491  0.4146639880002387  0.0235350412075657
  0.8266731078509536  0.5853360119997612  0.9764649777924325
  0.3266730778509510  0.0853360119997612  0.5235350222075675
  0.6733268921490464  0.9146639880002388  0.4764649477924371
  0.8266731078509536  0.4146639880002387  0.4764649477924371
  0.1733269221490491  0.5853360119997612  0.5235350222075675
  0.6733268921490464  0.0853360119997612  0.9764649777924325
  0.3266730778509510  0.9146639880002388  0.0235350412075657
  0.1663461659446455  0.1296508918501650  0.2121544829734355
  0.8336538630553534  0.8703490931498372  0.7878455020265669
  0.3336538340553545  0.3703490931498372  0.7121544979734331
  0.6663461369446466  0.6296509068501628  0.2878455020265668
  0.8336538630553534  0.1296508918501650  0.2878455020265668
  0.1663461659446455  0.8703490931498372  0.7121544979734331
  0.6663461369446466  0.3703490931498372  0.7878455020265669
  0.3336538340553545  0.6296509068501628  0.2121544829734355
```

4,4T286 carbon structure (.vasp file):

```
4,4T286
   1.00000000000000
     2.4873149711356057    0.0000000000000000   -0.0251552576308053
     0.0000000000000000    4.7054997020498393    0.0000000000000000
    -0.5326216452320398    0.0000000000000000    3.9613400002049128
   C
     8
Direct
  0.0319394715298125  0.1186819705267294  0.3686209931313367
  0.9680605544701895  0.8813180294732704  0.6313790068686632
  0.4680605244701871  0.1186819705267294  0.1313790068686633
  0.5319394455298105  0.8813180294732704  0.8686209931313368
  0.2268332473957587  0.4083699476614663  0.5584067227058872
  0.7731667526042412  0.5916300823385363  0.4415932772941129
  0.2731667526042413  0.4083699476614663  0.9415932772941128
  0.7268332473957588  0.5916300823385363  0.0584067227058872
```

Silica phase with bbi topology (.vasp file):

```
SiO2
   1.00000000000000
     7.1754458062286517    0.0001751518080634   -0.0001675296165496
     0.0000865595645469    7.8475985746848815    0.0000399378823383
     0.0000859933650461   -0.0001078276693343   10.3608869664822905
   O    Si
    24    12
Direct
  0.8539353311206762  0.5195851954045059  0.6738098035077780
  0.1459734567913770  0.4803045088320549  0.1737579903278549
  0.1459698875430020  0.5195760652103930  0.8262152289998116
  0.8539967064381245  0.4803275752507299  0.3262730971520753
  0.3539553585042006  0.0196858549248789  0.6737641866166260
  0.6459796014098630  0.9803879238272084  0.1737929010922429
  0.6459365555042748  0.0196681446779238  0.8262416850571639
  0.3539644388301180  0.9804411405023972  0.3262248214652747
  0.8889057763667366  0.2754793832368541  0.8526349861881904
  0.1112313355048291  0.7244023156552897  0.3525314609000603
  0.1109660511424879  0.2754665489757860  0.6473857373320352
  0.8887652548382819  0.7243851020725544  0.1474745394544933
  0.3887466550700209  0.7756000240469335  0.8525339715981421
  0.6110690371843649  0.2244967012770238  0.3526258234687665
  0.6111433756609951  0.7755923219290335  0.6474618407379678
  0.3889313992517873  0.2245008520130773  0.1473882516209667
  0.9999294249346422  0.9830316602761908  0.7499852111585197
  0.9999854810592375  0.0167874302637034  0.2499752047535164
  0.4999483615961424  0.4831770842383216  0.7499915587862968
  0.4999991930340458  0.5169328412678362  0.2499969439889620
  0.0831052856547579 -0.0001592256104303  0.4999983665035807
  0.9168070304700541 -0.0001694798949817 -0.0000186220887934
  0.5831287375641159  0.5001115548946634  0.4999888182580289
  0.4168213253628203  0.5001038090329678  0.0000076511722343
  0.9999492782513471  0.3980998847153954  0.7500144286926166
 -0.0000083276293611  0.6017599900712108  0.2500145114402422
  0.4999485041980247  0.8982116962757818  0.7499991932065901
  0.4999884699099095  0.1018778138851050  0.2500085671572184
  0.1386175169279983  0.0683138864900426  0.6423460101574161
  0.8612595668549948  0.9315580456911353  0.1423463683176060
  0.8612755603072568  0.0683110416287729  0.8576510110421995
  0.1386950639491298  0.9315644332920786  0.3576269931004000
  0.6386660394600356  0.5684076328589323  0.6423744019732786
  0.3613180726137070  0.4316724649169624  0.1423330550823489
  0.3612493194459633  0.5684060391526709  0.8576306365358838
  0.6386654179904073  0.4316722555567351  0.3576680305469860
```

Supplementary movie

The movie shows the energy profile for the tridymite $\leftrightarrow$ keatite transition and the corresponding structural changes during the transition in the [010] and [001] projections.
